# Reply to Ducreux, B.; Fauque, P. Comment on “Cannarella et al. DNA Methylation in Offspring Conceived after Assisted Reproductive Techniques: A Systematic Review and Meta-Analysis. *J. Clin. Med.* 2022, *11*, 5056”

**DOI:** 10.3390/jcm12010254

**Published:** 2022-12-29

**Authors:** Rossella Cannarella, Andrea Crafa, Aldo E. Calogero

**Affiliations:** Department of Clinical and Experimental Medicine, University of Catania, 95123 Catania, Italy

We thank our French colleagues for commenting [1] on our recently published article, which aims to understand whether there are differences in the methylation rates between offspring conceived using assisted reproducing techniques (ART) and those conceived spontaneously [2]. To accomplish this, we analyzed methylation in different tissues (placenta, cord blood, buccal smear, and peripheral blood) at different ages.

The methodology used to retrieve the articles is reported in the appropriate section of the manuscript, where all the criteria used are meticulously explained and follow the PRISMA guidelines [3]. Accordingly, the eligibility criteria (Population, Exposure, Comparator, Outcome, Study design), information sources, search strategy, and key string are explained in detail. We intentionally preferred to use the key term “epigenetic” and not “methylation” to make our search strategy as inclusive as possible. This approach was effective, as we were able to include up to 50 articles. All included articles underwent qualitative synthesis (systematic review), while only 12 articles could be meta-analyzed.

Several genes could be analyzed in our study, such as *H19* CCCTC-binding factor 3 (CTCF3), *H19* CCCTC-binding factor 6 (CTCF6), *Potassium Voltage-Gated Channel Subfamily Q Member 1* (*KCNQ1OT1*), *Paternally-expressed gene 3* (*PEG3*), and *Small Nuclear Ribonucleoprotein Polypeptide N* (*SNRPN*). For each of them, we divided the main analysis in several sub-analyses, according to the specific tissue used. This statistical approach makes it possible to evaluate the results of each sub-analysis individually, thus overcoming the problem of heterogeneity due to the pooling of different tissues. Regarding intra-tissue heterogeneity, this is clearly calculated by the I^2^ test that measures inter-study heterogeneity of the sub-analyses. Furthermore, to overcome inter- and intra-study heterogeneity, we used the standardized mean difference (SMD) (and not the mean difference, as used by Barberet and colleagues [4]). This approach is more statistically sound as the SMD is usually chosen when the methods used to measure outcomes differ across studies [5]. Indeed, the protocol used to measure DNA methylation is not the same in the various studies included in this meta-analysis and thus the SMD is statistically more accurate.

Concerning the issue of pooling data from different ages together, we disagree with our colleagues on this point, as a meta-analysis calculates the difference between two groups, whose ages are matched. The comparison could not be made if the two groups (patients and controls) were not age matched, but this was not the case. Therefore, the results of the sub-analyses are unbiased for both the tissue and the age issues.

Our French colleagues disagree with our exclusion of the ICSI samples of Sakian et al. [6] and Wong et al. [7] from the analysis. However, we confirm that these article should not be included, as their addition would compromise the reliability of the analysis. Indeed, the same control group (for IVF and for ICSI study sub-groups) would be used for both studies, and from a statistical point of view, this is not correct, as the data from the controls would be overweighed. This mistake is actually called *unit-of-analysis error* or *double counting*, and leads to overstatement of the evidence [8]. Unfortunately, this mistake is present in the meta-analysis of our colleagues [4].

We thank our colleagues for their comments regarding the studies by Puumala et al. [9], Choux et al. [10], and Sakian et al. [6]. For the outcome *H19* CTCF3, we modified the spontaneous conception data from the study by Pumala and colleagues [9], and added the data from cord blood from Choux and colleagues [10] (Figure 2) [2]. Also, we modified the sample size of the study by Sakian and colleagues [6] for *H19* CTCF6 (Figure 3) [2]. However, even when repeating the analysis and including their suggestions, the overall results do not change.

In conclusion, we appreciate the interest of our colleagues in reading our article [2]. We here fully respond to their comments, demonstrating the reliability of our approach. Finally, we would like to note that there is no perfect search strategy, as using different key terms can affect the articles retrieved. That is why different meta-analyses can arrive at different results which, in some respects, complement each other.
